# Prenatal-Onset Recessive Titinopathies: Clinical Spectrum, Genotype–Phenotype Correlations, and Outcomes

**DOI:** 10.3390/diagnostics16111723

**Published:** 2026-06-03

**Authors:** Yu Zheng, Mengmeng Shi, Yilin Zhao, Teresa Cheuk Yan Chung, Matthew Hoi Kin Chau, Zirui Dong, Yvonne Ka Yin Kwok, Hoi Wan Angel Kwan, Josephine Shuk Ching Chong, Tak Yeung Leung, Tsz Kin Lo, Kwong Wai Choy, Yanyan Zhang, Ye Cao

**Affiliations:** 1 Department of Obstetrics and Gynaecology, The Chinese University of Hong Kong, Hong Kong SAR, China; haleyzheng@cuhk.edu.hk (Y.Z.); mengmengshi@cuhk.edu.hk (M.S.); zhaoyilin@link.cuhk.edu.hk (Y.Z.); tchung19g@link.cuhk.edu.hk (T.C.Y.C.); matthewchau@cuhk.edu.hk (M.H.K.C.); elvisdong@cuhk.edu.hk (Z.D.); kky254@ha.org.hk (Y.K.Y.K.); angelkwan@cuhk.edu.hk (H.W.A.K.); tyleung@cuhk.edu.hk (T.Y.L.); richardchoy@cuhk.edu.hk (K.W.C.); 2 Hong Kong Hub of Paediatric Excellence, The Chinese University of Hong Kong, Hong Kong SAR, China; 3 Shenzhen Research Institute, The Chinese University of Hong Kong, Shenzhen 518000, China; 4 Fertility Preservation Research Centre, Department of Obstetrics and Gynaecology, The Chinese University of Hong Kong, Hong Kong SAR, China; 5 The Chinese University of Hong Kong-Baylor College of Medicine Joint Center for Medical Genetics, Hong Kong SAR, China; chongsc@cuhk.edu.hk; 6 Department of Paediatrics, The Chinese University of Hong Kong, Shatin, Hong Kong SAR, China; 7 Department of Obstetrics and Gynaecology, Princess Margaret Hospital, Hong Kong SAR, China; lotk@ha.org.hk; 8 Multi-Omics Center, MGI Tech, Shenzhen 518083, China

**Keywords:** *TTN*, *TTN*tv, titinopathy, prenatal diagnosis, fetal akinesia, arthrogryposis, fetal demise, cardiomyopathy, myopathy

## Abstract

**Background/Objectives:** Recessive titinopathies caused by biallelic *TTN* truncating variants (*TTNtvs*) present a clinically heterogeneous spectrum from fetal demise to late-onset slowly progressive distal muscular dystrophy. Prognostic counseling is challenging due to the vast size of the *TTN* gene, complex splicing patterns, and differential expression throughout developmental stages and tissues. This paper aims to delineate the regional genotype patterns and clinical characteristics of recessive titinopathies described from the prenatal period onwards to inform genotype–phenotype associations and genetic counseling. **Methods:** We analyzed clinical and genetic data from a prenatal-onset cohort with biallelic *TTNtvs* from both previously reported cases and novel cases from our center. To characterize the regional distribution of biallelic variants within this specific cohort, a two-dimensional scatter plot was utilized to map variants onto 10 biological regions (R1–R10) and 55 analytical units (U1–U55). We also performed Fisher’s exact tests on the subset of 50 cases with confirmed survival records to evaluate statistically significant associations between biallelic regional or percent spliced-in (PSI) thresholds combinations and severe clinical endpoints (intrauterine demise or death before 5 years). **Results:** A total of 96 prenatal cases from 76 unrelated families were analyzed. Decreased fetal movement was the most commonly reported symptom, observed in 81.3% (78/96) of cases, which was followed by arthrogryposis in 45.8% (44/96) and amniotic fluid volume abnormalities in 35.4% (34/96). Additionally, of the 95 cases with known pregnancy outcomes, 25.3% (24/95) resulted in termination and 11.6% (11/95) resulted in intrauterine demise (IUD), while 63.2% (60/95) reached birth with over 16.7% (10/60) being preterm. Among 60 live-born infants, severe postnatal morbidity was high: 45.0% (27/60) experienced respiratory failure, and 33.3% (20/60) died before the age of five. In this cohort, 84.4% (81/96) of cases possessed at least one *TTN*tv in either the metatranscript-only or A-band regions. The most common biallelic changes involved *TTNtvs* in both the A-band and metatranscript-only regions, accounting for 35.4% (34/96) of cases, followed by metatranscript-only combined with I-band variants at 16.7% (16/96), regardless of the PSI score of exons. Overall, 83.3% (80/96) had ≥1 variant on low-PSI (<50%) exons, and 19.8% (19/96) had both alleles on these low-PSI exons. In the 50 patients with confirmed survival records, biallelic changes (excluding splice-site variants) affecting both high-PSI (>90%) exons were significantly associated with severe outcomes (intrauterine demise or death before 5 years; exact *p* = 0.015), whereas the metatranscript-only plus I-band combination conferred a significantly lower risk of lethality before 5 years of age (exact *p* = 0.001). **Conclusions:** Our findings add to the accumulating evidence that *TTNtvs* on low PSl exons or metatranscript-only regions are frequently observed among reported prenatal-onset recessive titinopathy. Health surveillance for heterozygous carriers among family members is warranted due to the substantial risk for adult-onset dilated cardiomyopathy and peripartum cardiomyopathy.

## 1. Introduction

Titin, encoded by the giant *TTN* gene, is the largest known human protein and plays a crucial role in sarcomere development, structure, signaling and myofibrillar stability during muscle contraction and relaxation [1]. The N-terminus of titin is embedded in the Z-disk of the sarcomere. The remainder of the molecule is divided between an elastic I-band region and a thick filament-binding A-band region with the C-terminus embedded in the M-band region [2]. The human *TTN* gene contains 364 exons spanning 283 kb; 363 of these are incorporated into the full-length metatranscript isoform (NM_001267550.1). The *TTN* gene has extensive alternative splicing generating multiple isoforms that differ in size and spring properties: the principal long cardiac isoform N2BA (NM_001256850.1), the soleus/skeletal long isoform N2A (NM_133378.4), and other short isoforms. Exonsnot represented in major adult skeletal or cardiac muscle isoforms are known as “metatranscript-only exons” [3].

Titinopathy is a group of hereditary myopathies that result from pathogenic variants in the *TTN* gene, predominant truncating variants (*TTNtvs*) (OMIM: 188840) with both dominant and recessive inheritance patterns. *TTNtvs* primarily affect the structural role of the protein via haploinsufficiency and dominant negative effects; these mechanisms impair sarcomere integrity and compromise the contractile capacity of cardiomyocytes [4]. Heterozygous *TTNtvs* commonly cause autosomal dominant dilated cardiomyopathy (DCM) with late-onset and age-dependent penetrance. Truncating variants in the *TTN* gene are a major genetic cause of peripartum cardiomyopathy (PPCM). The prevalence of *TTNtvs* in women with PPCM is significantly higher than in a general population (*p* = 1.3 × 10^−7^), and it is similar to that observed in cohorts of patients with DCM [5,6]. *TTNtvs* are also encountered in ~1% of the general population where they may be asymptomatic due to variable expressivity influenced by variant position, exon usage, and genetic modifiers [3]. However, recent advancements in basic science have unveiled additional molecular roles of Titin beyond sarcomere stability and contraction, particularly highlighting its involvement in cellular signaling [7].

Recessive titinopathies caused by biallelic *TTN* pathogenic variants represent a clinically heterogeneous spectrum ranging from fetal demise to mild weakness manifesting in adulthood [8,9]. Prenatal diagnosis and counseling are often hindered by the extreme clinical heterogeneity, non-specific ultrasound findings, and the intricate genotype–phenotype correlations modulated by *TTN*’s extensive alternative splicing. While variant location is recognized as important, a systematic, high-resolution method to visualize and interpret the complex regional combinations of biallelic variants in relation to specific severe clinical endpoints (e.g., respiratory failure, early-onset cardiomyopathy etc.) is not well established [10,11,12,13,14]. Current evidence is fragmented across case reports [11,15,16] and small series [17,18,19], limiting the ability to provide accurate prognostic information and tailored genetic counseling to families facing a prenatal diagnosis.

Here, we characterize the clinical features and regional genotype patterns of prenatal-onset recessive titinopathy. Using a two-dimensional scatter plot to map biallelic variants of their regional distribution across the *TTN* gene, we aimed to correlate these combinations and severe clinical outcomes. This retrospective study integrates original cases from our prenatal diagnosis center with a review of published literatures.

## 2. Materials and Methods

### 2.1. Patient Recruitment

This retrospective study integrated original cases from our prenatal diagnosis center (during the 2020–2025 period) and cases identified through a literature review. The final cohort consisted of 96 prenatal cases of recessive titinopathy from 76 unrelated families, including two cases from our center and 94 cases from the literatures. The study protocol was approved by the Joint Chinese University of Hong Kong-New Territories East Cluster Clinical Research Ethics Committee (CREC Ref. No.: 2016.713). Written informed consent was obtained from the families of the original cases.

### 2.2. Genetic Work-Up

For the two novel cases, trio-based genetic analysis was performed. Fetal DNA was extracted from amniotic fluid, and parental DNA was obtained from peripheral blood. Genomic analysis included rapid aneuploidy detection via Quantitative Fluorescence PCR (QF-PCR), chromosomal microarray analysis (CMA) or karyotyping for copy number variations (CNVs), and trio genome sequencing (GS). Variants were detected using established and in-house bioinformatic pipelines for single nucleotide variants (SNVs), insertions/deletions (Indels), and CNVs [20,21,22]. Variant interpretation adhered to the American College of Medical Genetics and Genomics (ACMG) and the Association for Molecular Pathology (AMP) guidelines [23].

### 2.3. Literature Review

The medical literature was searched in PubMed (*TTN*[tiab] OR Titin[tiab] OR titinopathy*[tiab]) AND (biallelic[tw] OR “compound heterozygous”[tw] OR homozygous[tw] OR recessive[tw] OR “in trans”[tw]) AND (prenatal[tw] OR fetal[tw] OR fetus[tw] OR intrauterine[tw] OR antenatal[tw] OR “in utero”[tw] OR pregnancy[tw] OR gestation[tw] OR obstetric[tw] OR trimester[tw] OR “gestational age”[tw] OR “maternal”[tw] OR congenital[tw] OR “birth”[tw] OR newborn[tw] OR neonate[tw] OR “fetal hydrops”[tw] OR “polyhydramnios”[tw] OR “oligohydramnios”[tw] OR arthrogryposis[tw] OR akinesia[tw] OR “fetal akinesia”[tw] OR “fetal movement”[tw] OR “prenatal diagnosis”[tw] OR “prenatal ultrasound”[tw]; the last search was undertaken on 31 March 2026). Reviews, meta-analyses, editorials, and studies that do not present original, individual-level patient data will be excluded. Within included studies, individual cases will be excluded from data synthesis if they lack an explicit documentation of prenatal phenotypic abnormalities (e.g., only described as “congenital” or “early-onset” without specification). Cases with incomplete genetic diagnostic information (e.g., unreported specific variants, absence of segregation data for recessive inheritance) will also be excluded.

Based on our literature review, 20 publications reporting at least one case with prenatal-onset recessive titinopathies were included. In total, 94 cases from 74 unrelated families were collected from the literature. Clinical details (prenatal phenotypes, gestational age of onset, pregnancy outcomes, postnatal severity, last check-up age and death age) were collected. Biallelic *TTN* variants identified in these cases were collected. Each variant was annotated according to Ensembl Variant Effect Predictor (VEP, https://asia.ensembl.org/info/docs/tools/vep/index.html, accessed on 2 April 2026) and the Titin Variants in Dilated Cardiomyopathy database (https://www.cardiodb.org/titin/titin_transcripts.php, accessed on 2 April 2026). The submissions of variant curation in ClinVar (https://www.ncbi.nlm.nih.gov/clinvar/, accessed on 2 April 2026) were also annotated if they were available.

### 2.4. Phenotype Data Analysis

Prenatal phenotypic data, including the specific manifestations and their gestational age at onset, were collected. All recorded abnormalities were categorized into ten distinct groups: (1) abnormal fetal movement, (2) contractures, (3) fetal hydrops/edema, (4) intrauterine growth restriction (IUGR), (5) cardiovascular anomalies, (6) fetal effusions, (7) micrognathia/retrognathia, (8) increased nuchal translucency (NT), (9) amniotic fluid (AF) abnormalities, and (10) other findings. The prenatal phenotypes and postnatal severity data of this study cohort were visualized using stacked bar charts. Pregnancy outcomes were visualized using bar charts. 

### 2.5. Genotype Data Analysis

Given the extraordinary size of the *TTN* gene and the heterogeneous distribution of disease-causing variants, to enable a regional analysis of biallelic variant distributions, we categorize the *TTN* variants reported in this cohort using a two-tier framework. Tier 1: Functional Region (R). The *TTN* coding sequence was partitioned into 10 contiguous, biologically coherent regions (R1-R10) based on sarcomere domain architecture, percent spliced in (PSI) score of exons, and established clinical association data summarized in the database Titin Variants in Dilated Cardiomyopathy (https://www.cardiodb.org/titin/titin_transcripts.php, accessed on 2 April 2026). Region definitions and exon ranges are detailed in Table 1. While the primary partitioning was guided by sarcomere domain architecture and exon splicing kinetics, we acknowledge that the discrete boundaries of biological regions do not always align perfectly with individual exon structures; consequently, a minority of exons were assigned to the nearest adjacent region to maintain analytical continuity. Tier 2: Analytical Unit (U). To enable unbiased visualization across the gene, we subdivided each region into analytical units of 5–7 consecutive exons, yielding 55 total units (U1–U55). Each unit is uniquely nested within one primary region (Supplementary Table S2). This range was selected to ensure the complete coverage of all band regions without crossing functional boundaries while maintaining consistent unit sizes for visualization. Because every unit is nested within a single region, the unit size does not affect region-level genotype–phenotype conclusions. Each biallelic genotype was represented as a coordinate pair (U_α_, U_β_) on a two-dimensional scatter plot. This coordinate system enables the visualization of variant combinations and facilitates the identification of regional patterns associated with specific phenotypic outcomes that would be obscured in traditional linear gene maps.

### 2.6. Statistical Analysis

The cases with definitive survival records were included in the further analysis to examine statistically significant associations between biallelic regional combinations and severe clinical endpoints (intrauterine demise or death before 5 years). Those who underwent termination of pregnancy (TOP) or were lost to follow-up before 5 years with unknown subsequent vital status were excluded. Case no. 60 was excluded from analysis, because the death of this fetus is highly suspected to twin-to-twin transfusion (recipient twin). The remaining analytic cohort comprised 50 patients classified into two groups: severe events (IUD or confirmed death before 5 years, *n* = 30) and confirmed survival beyond 5 years (n = 20). Patients were stratified by the presence or absence of specific biallelic *TTN* variant combinations across sarcomere regions (M-band, A-band, I-band, Z-band, and metatranscript-only regions) or by percent spliced-in (PSI) score. Associations between each genotype group and clinical outcome were evaluated using two-sided Fisher’s exact tests; exact odds ratios (ORs) with 95% confidence intervals (CIs) were derived from the conditional maximum likelihood estimate implemented in the fisher.test function (R stats package). When zero cells preclude finite interval estimation, the exact confidence limit is reported as unbounded. Analyses were performed in RStudio (Posit team (2025). RStudio: Integrated Development Environment for R.Posit Software, PBC, Boston, MA. URL http://www.posit.co/, version 2026.01.1+403); a nominal *p*-value < 0.05 was considered significant. As this was an exploratory analysis involving multiple predefined comparisons, *p*-values were reported without adjustment for multiple testing.

## 3. Results

### 3.1. Two Affected Families from Our Center

Family 1

A 42-year-old woman presented at 12^+3^ gestational weeks (GW) with a fetus showing increased nuchal translucency (NT) of 4.4–4.5 mm and dilated bilateral jugular lymphatic sacs (2–3 mm) at 14 GW. The non-invasive prenatal test (NIPT) was negative [24]. Fetal echocardiography at 17^+2^ GW revealed a cardiothoracic ratio of 0.61, globular dilation of the right ventricle (RV), poor global cardiac contractility, and limited inflow through a structurally normal tricuspid valve. The pulmonary valve showed severely restricted mobility, indicating critical pulmonary stenosis. Additional findings included subcutaneous edema of the scalp, neck, and upper thorax along with a clubbed left foot. Pregnancy was terminated at 18 GW. Postmortem examination confirmed RV dilation and an enlarged left ventricle with no structural abnormalities noted. Genomic sequencing identified compound heterozygous variants in the *TTN* gene: a maternally inherited frameshift variant (c.1732dupG, p.Glu578GlyfsTer22, located in the Z-disk with 51% PSI score) classified as Likely Pathogenic (criteria PVS1+PM2_supporting) and a paternally inherited variant (c.94855C>T, p.Arg31619Ter, located in the A-band with 100% PSI score) classified as Pathogenic (PVS1+PM2_supporting+PP4).

Family 2

A 39-year-old woman was admitted at 26 and 29 GW for reduced fetal movement. At 29 GW, polyhydramnios developed with an amniotic fluid index (AFI) of 26 cm. Ultrasound at 30^+4^ GW showed an AFI of 17.9 cm, mild prenasal edema, a persistently closed mouth, right positional clubfoot, splaying of bilateral toes, and hyperflexion of the left wrist. Follow-up ultrasound at 34^+6^ GW revealed normal fetal growth and amniotic fluid volume with observed fetal swallowing and breathing movements; however, no limb movement was noted, and the left hand remained fixed in a flexed position. The fetus showed no cardiac abnormalities or hydrops. The infant was delivered with cyanosis and irregular breathing, dying 15 days later. Physical examination revealed a high-arched palate, low-set ears, severe retrognathia, right choanal atresia, and joint contractures affecting nearly all limb joints as well as absent flexion skin creases and bilateral ankle contractures. Genomic sequencing identified compound heterozygous variants in the *TTN* gene in the fetus: a maternally inherited frameshift variant (c.38876-2A>C, located in the meta transcript-only exon with 2% PSI score), classified as Pathogenic (criteria PVS1+PM3+PM2_supporting), and a paternally inherited frameshift variant, c.951delC (p.Ile318SerfsTer42, located in the Z-disk with 100% PSI score), which was classified as Likely Pathogenic (PVS1+PM2_supporting).

The clinical and genetic information of these cases are summarized in Table 2.

### 3.2. Clinical Spectrum, Outcomes, and Genotype–Phenotype Correlations of Recessive Titinopathies

A total of 20 publications reported cases of prenatal-onset recessive titinopathies, encompassing 94 cases from 74 unrelated families. Including two novel cases, this study analyzed 96 cases from 76 unrelated families. Detailed clinical and genetic profiles of these cases are provided in Supplementary Table S1.

Prenatal Clinical Spectrum

**Figure 1:**
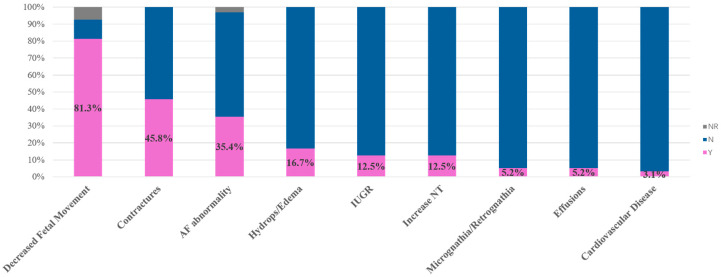
Reported prenatal clinical spectrum of recessive titinopathies. IUGR, intrauterine growth restriction; NT, nuchal translucency; NR, not reported; N, no; Y, yes.

According to this cohort, decreased fetal movement during the second trimester was the most common, which was reported in 81.3% (78/96) of cases (Figure 1). Limbs contractures were the second most common finding, presenting 45.8% (44/96) of fetuses, including a fixed flexion or extension deformities of the joints, generalized joint immobility, and a limited range of motion. Amniotic fluid volume abnormalities, either oligohydramnios or polyhydramnios, were also frequently noted, affecting 35.4% (34/96). Less common findings included fetal hydrops in 16.7% (16/96), IUGR and increased NT, which were both reported in 12.5% (12/96). Congenital heart malformations are not frequently observed in this cohort, presenting in only 3.1% (3/96).

Pregnancy Outcomes

**Figure 2:**
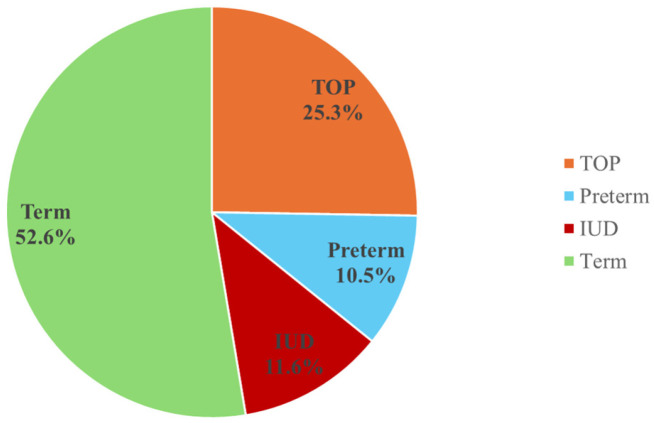
Pregnancy outcomes in prenatal-onset recessive titinopathies (*n* = 95). TOP: termination of pregnancy; Preterm: preterm birth; IUD: intrauterine death; Term: term birth.

Outcome data were analyzed for 95 of the 96 cases in this cohort with one case excluded due to incomplete pregnancy outcome records (Figure 2). Then, the pregnancy outcomes were further grouped into four categories: term birth, preterm birth, intrauterine fetal demise (IUD), and termination of pregnancy (TOP). Termination of pregnancy was performed in 25.3% (24/95) which was usually with fetal anomalies, such as multiple contractures or fetal hydrops. Notably, IUD occurred in as high as 11.6% (11/95) of pregnancies. Preterm birth (<37 weeks’ gestation) was observed in 10.5% (10/95) of cases, which was potentially precipitated by severe fetal compromise or associated obstetric complications. Approximately half (52.6%, 50/95) of the pregnancies resulted in term live births, which will be further characterized in the subsequent postnatal follow-up analysis.

Postnatal Severity of Prenatal-Onset Recessive Titinopathies

**Figure 3:**
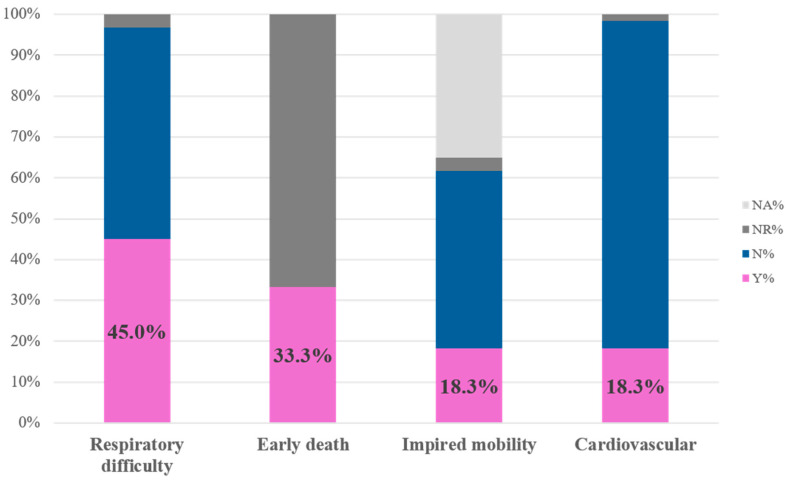
Postnatal severity of prenatal-onset recessive titinopathies. NA, not applicable; NR, not reported; N, no; Y, yes.

In this cohort, 60 live births were available (63.2%, 60/95) for postnatal follow-up. All 60 cases (100%) exhibited features consistent with a severe congenital form of titinopathy, which universally manifested as severe hypotonia and profound motor impairment in the neonatal period. To assess the outcome severity, we analyzed four predefined clinically critical endpoints among these children (Figure 3). Respiratory failure, requiring assisted ventilation (non-invasive or invasive) at any point during follow-up, was the most common, which affected 45.0% (27/60). This indicated that nearly half of live births would experience life-threatening respiratory compromise, which may correlate with the high early childhood mortality (before age 5 years) of 33.3% (20/60). Severe motor disability, defined as the inability to achieve independent sitting, was present in 18.3% (11/60) of the cohort. Cardiac anomalies, including cardiomyopathy or structural defects, were identified in 18.3% (11/60), occurring less frequently than skeletal muscle-related issues.

Regional Distribution of Biallelic Variants Reveals High-Risk Genotypic Patterns

A total of 111 unique variants (Supplementary Table S3) were identified in this cohort. Of these, 108 (97.2%) were truncating variants (nonsense, frameshift, or canonical splice site change). Of three missense variants, two (c.56329A>G; c.36285C>G) had high SpliceAI scores (>0.8) predictive of splicing disruption, therefore grouping them as truncating variants in the downstream analyses. The remaining one missense variant (c.85328T>A) occurred in trans with a truncating variant (NM_001267550), which was segregated with multiple affected individuals in the family. While most variants are rare in population databases, 9.91% (11/111) cluster exclusively in a single exon 327 on the A-band (R8/unit 49), which is the largest exon (over 17 kb) of the *TTN* gene. Notably, nearly one third (33/111, 29.7%) were mapped to the A-band, and 27.0% (30/111) were in meta-only transcripts. Variants in other *TTN* regions were distributed as follows: I-band: 26.1% (29/111), M-band: 11.7% (13/111), near-Z-disk: 4.51% (5/111), and Z-disk: 0.90% (1/111). Furthermore, 73.0% (81/111) were expressed in N2BA transcripts (NM_001256850.1), while 51.4% (57/111) were found in N2B transcripts (NM_003319.4) and 73.0% (81/111) in the soleus/skeletal long isoform N2A (NM_133378.4).

**Figure 4:**
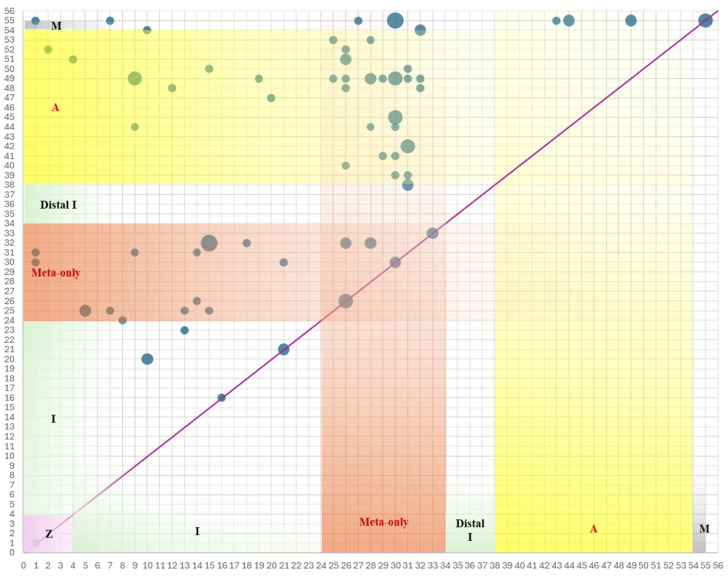
A 2D scatter plot visualization of biallelic *TTNtvs* in this cohort. *X*-axis: position of the first allele (Allele 1) across virtual units (U1–U55); *Y*-axis: position of the second allele (Allele 2). Larger virtual unit (U) values correspond to the position of Allele 1. Each bubble represents a unique biallelic combination. Bubble size is proportional to the number of cases sharing that specific unit combination (i.e., the co-occurrence frequency of Allele 1 and Allele 2). The dashed diagonal line represents biallelic variants localized within the same virtual unit.

The 2D scatter plot visualization at the virtual unit (U) level illustrated the regional distribution of biallelic variants in this cohort (Figure 4) and across different phenotype cohorts (Supplementary Figures S1–S3). Notably, 84.4% (81/96) of cases possessed at least one *TTN*tv in either the metatranscript-only or A-band regions. The most common biallelic changes involved *TTNtvs* in both the A-band and metatranscript-only regions, accounting for 35.4% (34/96) of cases, followed by metatranscript-only combined with I-band variants at 16.7% (16/96) and metatranscript-only variants combined with other metatranscript-only variants at 11.5% (11/96), regardless of the percent spliced in (PSI) score of exons in the metatranscript-only or I-band regions.

The PSI score of the exons harboring these variants ranged widely from 0 to 100. All exons with variants in the A-band and M-band showed a PSI of 100%, whereas the exons of six variants in the I-band exhibited variable averages of 33% PSI, ranging from 6% to 78%. In contrast, the 14 metatranscript-only exons with 18 variants showed nearly 0% PSI with an average of 3% and a range of 0% to 7%. Overall, 83.3% (80/96) of this prenatal cohort had ≥1 variant on low-PSI (<50%) exons, and 19.8 (19/96)% had both alleles on these low-PSI exons. These findings underscore the clinical significance of variants that disrupt exons with low PSI in recessive *TTN* disorders, which are often overlooked, as truncated variants affecting these exons are frequently classified as variants of uncertain significance (VUSs) in ClinVar.

To evaluate whether the spatial distribution of biallelic truncating variants modulates clinical severity, we performed Fisher’s exact tests to examine the association between 12 predefined biallelic *TTN* regional combinations and severe clinical outcomes (IUD or death before 5 years) in 50 patients with definitive survival records (Supplementary Table S4). Among 12 predefined regional combinations, the metatranscript-only plus I-band genotype was significantly associated with a lower risk of severe outcomes (0/7 [0%] vs. 30/43 [69.8%] in others; exact *p* = 0.001, exact OR < 0.001, 95% CI 0.000–0.364), indicating reduced lethality within the context of substantial morbidity. Conversely, biallelic changes (excluding splice-site variants) affecting both high PSI (>90%) were significantly associated with severe outcomes (8/8 [100%] vs. 22/42 [52.4%]; exact *p* = 0.015, exact OR = ∞, 95% CI 1.322–∞). No other regional or PSI-based combination reached statistical significance (all *p* > 0.05). For groups with zero events in the exposed arm, exact odds ratios were formally infinite or zero, reflecting complete outcome separation in small samples; the corresponding finite confidence limit indicates the minimum detectable non-null effect (Supplementary Table S5).

## 4. Discussion

This study represents the largest cohort to date investigating prenatal-onset recessive titinopathy, including 96 prenatal cases from 76 unrelated families harboring 111 unique *TTN* variants. This provides insight into the early onset and severe spectrum of disease trajectory and genotype–phenotype correlations.

The phenotype spectrum of our prenatal cohort reflects a markedly severe clinical profile compared to late-onset, slowly progressive recessive titinopathy (MIM 608807,600334) [8]. Nearly 12% of the pregnancies ended in intrauterine fetal demise, highlighting the life-limiting nature of the disease in a significant subset. While 45% of live-born children experienced respiratory failure requiring assisted ventilation and 33% died before the age of five, these rates are significantly higher than those reported in postnatal cohorts, underscoring the severe trajectory of prenatal-onset recessive titinopathy. However, recessive titinopathy might not exhibit specific and detectable structural abnormalities in the first trimester other than non-specific soft markers. Decreased fetal movement (81%) and fetal arthrogryposis, usually bilateral or multiplex (46%), are common phenotypes that may become apparent in the second trimester or later. Structural cardiac abnormalities are rarely reported prenatally and are significantly lower than the 46% incidence of congenital and/or early-onset cardiac pathology noted in very young children [25]. This discrepancy suggests that the cardiac phenotype may evolve postnatally as hemodynamic changes occur after birth, or that subtle functional impairments (e.g., diastolic dysfunction) may be undetectable by standard fetal echocardiography yet predispose individuals to early heart failure. Ultimately, recessive titinopathy tends to manifest more as a skeletal myopathy, as biallelic *TTN* loss-of-function disrupts the mechanosensory and structural integrity of the sarcomere early in development, leading to a fetal akinesia deformation sequence.

Our regional analysis revealed biallelic genotype characteristics within this cohort of individuals with severe spectrum recessive titinopathy, indicating not all regions are related to equivalent clinical risk. Among the 111 unique *TTN *variants, A-band variants constituted 29.7% (33/111), with 9.9% (11/111) clustering specifically within exon 327 (proximal A-band; R8/Unit 49). Metatranscript-only region variants (R6/Units 25–33) accounted for 27.0% (30/111), with over half (53.3%, 16/30) in Unit 26 or Unit 30 (8 variants in each). In contrast, I-band variants (26.1%, 29/111) displayed a scattered distribution with no evident regional hotspot. At the case level (n = 96),84.4% (81/96) of cases possessed at least one *TTNtv* in either the metatranscript-only (69.8%, 67/96) or A-band regions (53.1%, 51/96). The most common biallelic changes involved *TTNtvs* in both the A-band and metatranscript-only regions, accounting for 35.4% (34/96) of cases, highlighting the critical, non-redundant function of these constitutive sarcomere domains. This was followed by metatranscript-only combined with I-band variants at 16.7% (16/96) and metatranscript-only variants combined with other metatranscript-only variants at 11.5% (11/96). Such non-uniform regional distribution was also observed in subgroups with early mortality and respiratory failure (Supplementary Figures S1–S3). This prenatal cohort revealed that the severe spectrum and outcome, such as early death and respiratory failure, are more likely associated with the presence of at least one truncating allele in the A-band, particularly in its distal section. These patterns offer an actionable framework for risk stratification in prenatal genetic counseling.

Metatranscript-only exons are typically spliced out in adult tissues; however, they may play a crucial role in early developmental isoforms [10,26]. Their involvement suggests that recessive titinopathy is not merely a “loss” of adult function but rather a disruption of developmentally specific splicing programs, leading to structural instability during critical periods of myofibrillogenesis. Therefore, the *TTN* truncated variants affecting these exons, especially those with low PSI, should be carefully evaluated and not necessary as variants of uncertain significance (VUSs). While metatranscript-only combined with other metatranscript-only variants were frequently reported in our cohort, no A-band combined with A-band variants were identified in this large prenatal cohort. It is suspected that biallelic A-band variants would disrupt all types of transcripts, leading to a complete loss of TTN protein function, which could be incompatible with life or normal embryonic development. This observation aligns with the biological role of these regions: the A-band constitutes the core of the thick filament and is constitutively expressed [27]. Truncations in this region are less tolerated due to the lack of redundancy in major isoforms (N2BA/N2A).

The regional distribution and exon PSI score of biallelic *TTN* truncating variants had an impact on the clinical severity in our cohort. Biallelic coding variants on high-PSI (>90%) exons were significantly associated with an increased risk of severe outcomes (exact *p* = 0.015, exact OR = ∞, 95% CI 1.322–∞), which was expected, as monoallelic *TTNtv* on high-PSI (>90%) exons has been known to cause dominant cardiomyopathy. Meanwhile, biallelic variants on low-PSI (<50%) exons (typically on meta-only or I-band regions) did not show a statistically significant reduction in severe outcome risk (*p* = 1.000, OR 0.804, 95% CI 0.147–4.694). However, the specific combination of meta-only + I-band variants was significantly associated with a less severe clinical course compared with other biallelic combinations in our cohort, using the composite endpoint of substantial morbidity (exact *p* = 0.001, exact OR < 0.001, 95% CI 0.000–0.364). This indicates that a single low PSI score alone cannot be used to downgrade the clinical significance of a variant; instead, milder phenotypes may arise from the interplay of regional location and other biological modifiers. Due to limited cases within each combination type, larger cohorts are needed to validate this observation and achieve adequate statistical power for rare combinations.

These correlations have immediate clinical utility. (1) In the prenatal setting, identifying the aforementioned genotypic patterns should prompt counseling about the very high risk of severe, life-limiting disease. (2) Family cascade testing is warranted, as the parents in our pedigrees who transmitted these A-band variants are obligate heterozygotes and therefore face a significantly increased lifetime risk of developing DCM. Notably, 61% (59/96) of cases in this cohort harbored at least one *TTN* variant located within exons with a PSI of 100% in the I-band or A-band regions. The typical onset of DCM associated with heterozygous *TTN* A-band variants occurs in mid-adulthood, with age-dependent penetrance, often in the fourth or fifth decade of life. In the context of our study, the parental cohort may not yet have reached this characteristic age of onset. Moreover, for reproductive carrier screening, this provides supporting evidence for reporting policies regarding recessive titinopathy-related carrier status [28]. The early detection of preclinical DCM in parents allows for timely intervention, shifting the focus from solely fetal prognosis to comprehensive family care. Recent large-scale registry studies indicate that women carrying *TTNtv* are particularly susceptible to peripartum cardiomyopathy (PPCM) [5,6]. Notably, *TTNtv*-positive women present with significantly lower left ventricular ejection fraction (LVEF 23.5% vs. 29% in controls, *p* = 2.5 × 10^−4^), suggesting a more severe phenotype upon hemodynamic stress [5]. Given that most parents in our cohort are obligate heterozygous carriers of *TTNtv*, these findings highlight the necessity of longitudinal cardiac surveillance. Specifically, family cascade testing and pre-pregnancy counseling are imperative for female relatives of affected individuals to monitor and manage the risk of PPCM.

This study has limitations. First, our cohort’s large reliance on previously published cases is subject to publication and ascertainment bias, which favors more severe phenotypes, and the sample size was limited for the regional analysis. Among cases carrying biallelic *TTN* truncating variants, those with severe fetal ultrasound findings, intrauterine demise, termination of pregnancy, or severe neonatal outcomes are likely overrepresented in the published literatures, while cases with relatively mild or later onset are likely unreported and cardiac status remains largely uncharacterized, precluding precise penetrance estimates for DCM risk. Third, the “PSI%” data used, derived from adult DCM hearts, may not fully reflect fetal or developmental splicing patterns in various muscle cells. Future prospective studies with systematic parental phenotyping and functional investigations into variant-specific effects on protein and splicing are needed.

## 5. Conclusions

Our findings add to the accumulating evidence that *TTNtvs* in low PSl exons or metatranscript-only exons are frequently observed in reported prenatal-onset recessive titinopathy. These variants are associated with severe outcomes including high rates of adverse pregnancy events and postnatal morbidity, especially when present in trans with an A-band variant. Additionally, these findings underscore the necessity of family cascade testing and longitudinal cardiac surveillance for heterozygous relatives, mitigating the lifetime risk of DCM and peripartum cardiomyopathy.

## Figures and Tables

**Table 1:** Definition of *TTN* regions and analytical units.

Region	Biological Significance and Percent Spliced in (PSI) Score *	Exon Range Based on Meta Transcript *	Unit Range
R1	N-terminal Z-disk	Exon 1–7	Unit 1
R2	Z-repeat domain	Exon 8–14	Unit 2
R3	Near Z-disk	Exon 15–28	Unit 3–4
R4	Proximal high PSI I-band	Exon 29–44	Unit 5–7
R5	Low PSI I-band	Exon 45–158	Unit 8–24
R6	Meta-only band	Exon 159–219	Unit 25–33
R7	Distal high PSI I-band	Exon 220–252	Unit 34–38
R8	Proximal high PSI A-band	Exon 253–327	Unit 39–49
R9	Distal high PSI A-band	Exon 328–358	Unit 50–54
R10	C-terminal M-band	Exon 359–364	Unit 55

* Refer to the database Titin Variants in Dilated Cardiomyopathy (https://www.cardiodb.org/titin/titin_transcripts.php, accessed on 2 April 2026).

**Table 2:** The two novel families with prenatal-onset recessive titinopathies from our center.

Case No.	Prenatal Phenotype (HPO ^1^)	Outcome	Allele/ Origin	Coordinate (hg19)	*TTN* Variant/Zygosity (NM_001267550.1)	Curation	ACMG ^2^	Band	Exon (PSI) ^3^/ Isoform ^4^
CU-1	12W+3: Increased nuchal translucency (HP:0010880) 14W: Distended jugular lymphatic sacs (HP:0025701) 17W: Cardiomegaly (HP:0001640) Right ventricular dilatation (HP:0005133) Valvular pulmonary stenosis (HP:0034350) Fetal skin edema (HP:0025672) Talipes equinovarus (HP:0001762)	Termination of Pregnancy Autopsy: Right ventricular dilatation (HP:0005133) Left ventricular dilatation (HP:4000141)	Allele 1/Maternal	2:179655502	c.1732dupG p.Glu578GlyfsTer22 /Het	Likely Pathogenic	PVS1 PM2_supporting	Z	11 (51%)/ N2BA, N2A, Nvx3
			Allele 2/Paternal	2: 179411203	c.94855C>T p.Arg31619Ter/Het	Pathogenic	PVS1 PM2_supporting PP4	A	343 (100%) Meta, N2BA, N2B, N2A, Nvx1, Nvx2, Nvx3
CU-2	26 W: Decreased fetal movement (HP:0001558) 29W: Polyhydramnios (HP:0001561) 30W+4: Fetal skin edema (HP:0025672) Talipes equinovarus (HP:0001762) Joint hypermobility (HP:0001382) 34W+6: Joint hypermobility (HP:0001382)	Term birth Respiratory insufficiency (HP:0002093) Neonatal death (HP:0003811) High palate (HP:0000218) Low-set ears (HP:0000369) Micrognathia (HP:0000347) Choanal atresia (HP:0000453) Joint contracture (HP:0034392)	Allele 1/Maternal	2:179517660	c.38876-2A>C/Het	Pathogenic	PVS1 PM3 PM2_supporting	Meta-only	201 (2%) Meta, N2BA, N2A, Nvx3
			Allele 2/Paternal	2:179659943	c.951delC p.Ile318SerfsTer42/Het	Likely Pathogenic	PVS1 PM2_supporting	Z	7 (100%) Meta, N2BA, N2B, N2A, Nvx1, Nvx2, Nvx3

^1^ HPO: Human Phenotype Ontology (https://hpo.jax.org/, accessed on 2 April 2026); ^2^ ACMG and score: standards and guidelines for the interpretation of sequence variants: a joint consensus recommendation of the American College of Medical Genetics and Genomics and the Association for Molecular Pathology (PMID 25741868); ^3^ PSI: percent spliced in; ^4^ Isoform: refer to Titin Variants in Dilated Cardiomyopathy (https://www.cardiodb.org/titin/titin_transcripts.php, accessed on 2 April 2026).

## Data Availability

The data are not publicly available because public access was not consented from the subjects in the study but could be available upon reasonable request from the corresponding author.

## Supplementary Materials

The following supporting information can be downloaded at https://www.mdpi.com/article/10.3390/diagnostics16111723/s1, Table S1: Clinical and genetic profile of cases with prenatal-onset recessive titinopathies. Table S2: *TTN* region definitions and exon ranges. Table S3: Unique *TTN* variants in this study cohort. Table S4: Summary of biallelic *TTN* combinations and severe clinical outcomes in patients. Table S5: Association between biallelic *TTN* combinations and severe clinical outcomes in patients. Figure S1: Enrichment of TTN Variants in Prenatal-Onset Recessive Titinopathies-Early Death & IUD Group (*n* = 31). Figure S2: Enrichment of *TTN* Variants in Prenatal-Onset Recessive Titinopathies-Respiratory Failure Group (*n* = 27). Figure S3: Enrichment of *TTN* Variants in Prenatal-Onset Recessive Titinopathies-Cardiovascular Disease Group (*n* = 14).

## Abbreviations

The following abbreviations are used in this manuscript:

ACMG	American College of Medical Genetics and Genomics
AF	Amniotic Fluid
AFI	Amniotic Fluid Index
AMP	Association for Molecular Pathology
CNV	Copy Number Variation
CREC	Clinical Research Ethics Committee
DCM	Dilated Cardiomyopathy
GS	Genome Sequencing
GWs	Gestational Weeks
HPO	Human Phenotype Ontology
IUGR	Intrauterine Growth Restriction
IUD	Intrauterine Fetal Demise
LVEF	Left Ventricular Ejection Fraction
NIPT	Noninvasive Prenatal Test
NT	Nuchal Translucency
PEFF	Pericardial Effusion
PPCM	Peripartum Cardiomyopathy
PSI	Percent Spliced In
QF-PCR	Quantitative Fluorescence Polymerase Chain Reaction
R	Region
RV	Right Ventricle
SNV	Single-Nucleotide Variant
TOP	Termination of Pregnancy
TTN	Titin
TTNtvs	Titin Truncated Variants
U	Analytical Unit
VEP	Variant Effect Predictor
VUSs	Variants of Uncertain Significance
