# Supplementary material for: Prenatal-Onset Recessive Titinopathies: Clinical Spectrum, Genotype–Phenotype Correlations, and Outcomes
Source: Diagnostics (Basel). 2026 Jun 3;16(11):1723. doi: 10.3390/diagnostics16111723 (PMC13257078; doi:10.3390/diagnostics16111723)
Supplement: Supplementary file 1 [file diagnostics-16-01723-s001.zip › Sup_Figures_Prenatal TTN_diagnostics_20260520.pptx]

## Slide 1
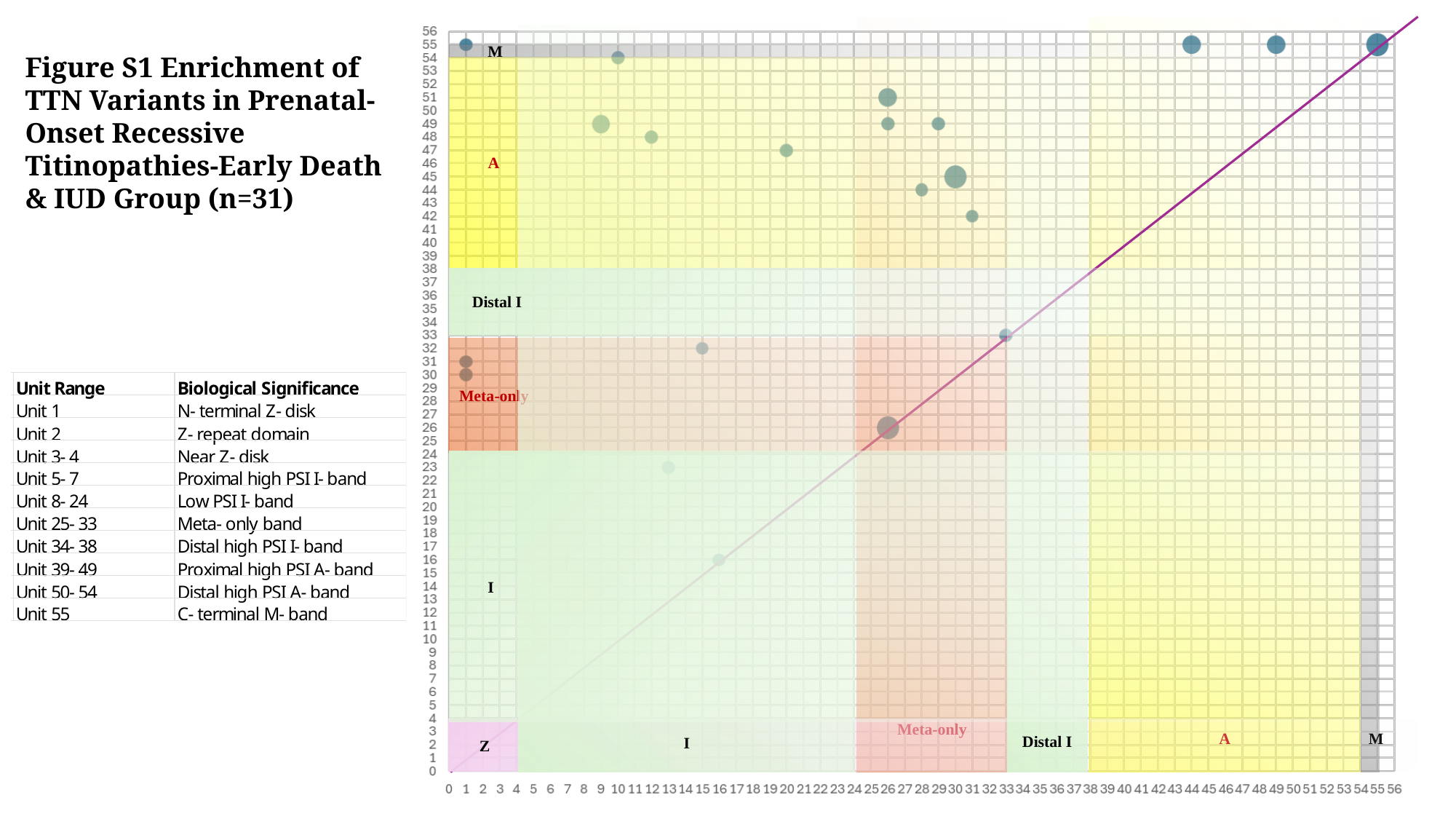

A
M
Meta-only
Distal I
I
Figure S1 Enrichment of TTN Variants in Prenatal-Onset Recessive Titinopathies-Early Death & IUD Group (n=31)
 M
 A
 Distal I
Meta-only
 I
 Z

## Slide 2
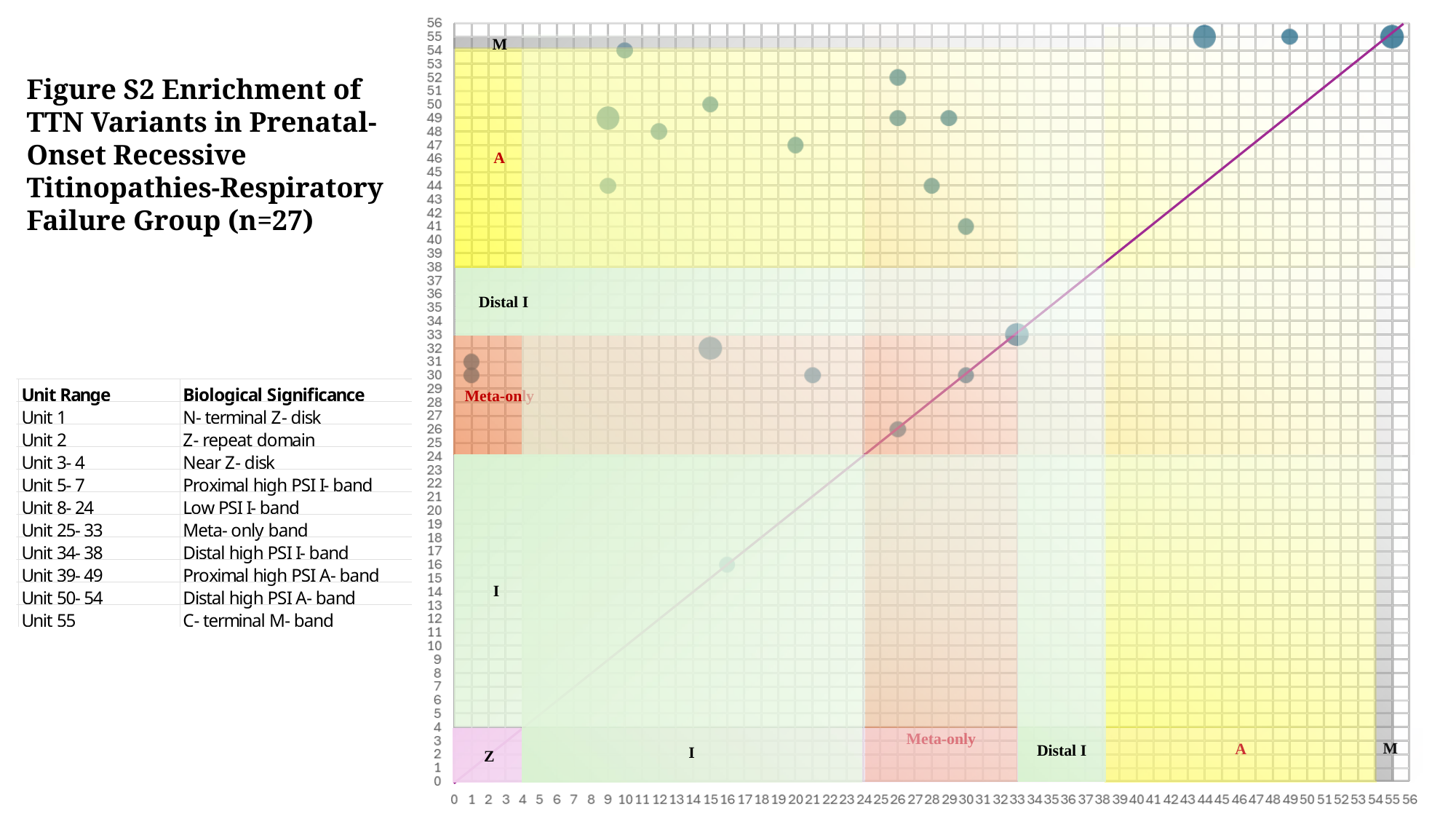

M
Meta-only
A
Distal I
I
 M
 A
Figure S2 Enrichment of TTN Variants in Prenatal-Onset Recessive Titinopathies-Respiratory Failure Group (n=27)
 Distal I
Meta-only
 I
 Z

## Slide 3
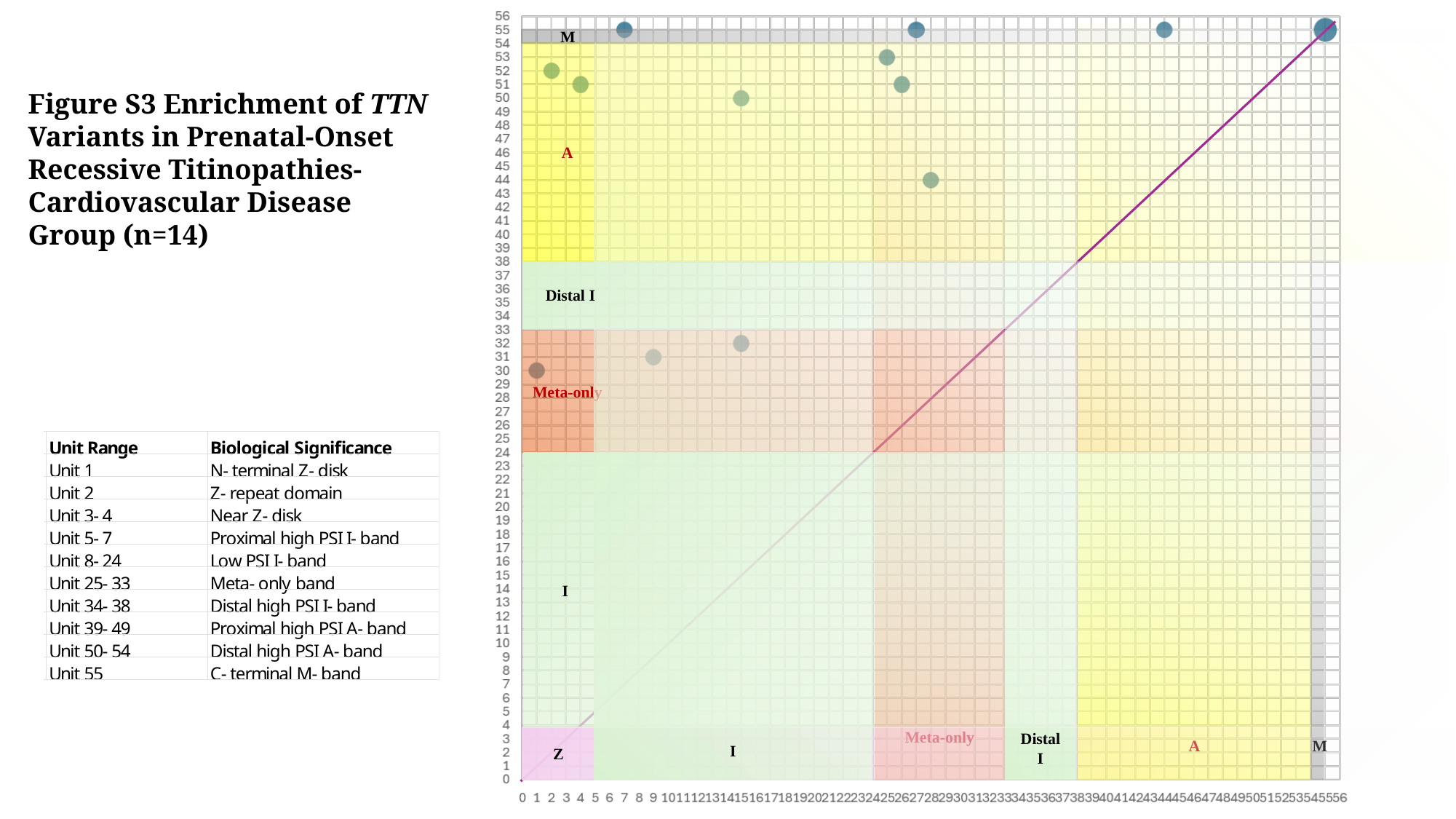

A
M
Meta-only
Distal I
 M
I
 A
Figure S3 Enrichment of TTN Variants in Prenatal-Onset Recessive Titinopathies-Cardiovascular Disease Group (n=14)
 Distal I
Meta-only
 I
 Z
